# Comparison of methods for estimating Young’s moduli of mortar specimens

**DOI:** 10.1038/s41598-024-65149-3

**Published:** 2024-06-20

**Authors:** Simon Schmid, Jithender J. Timothy, Elena Woydich, Jochen Kollofrath, Christian U. Grosse

**Affiliations:** Department of Materials Engineering, TUM School of Engineering and Design, Technical University of Munich, Franz-Langinger-Str. 10, Munich, 81245 Bavaria Germany

**Keywords:** Young’s modulus, Compression test, Ultrasound, Simulation, Computed tomography, Inverse problem, Materials science, Engineering, Civil engineering

## Abstract

Precisely estimating material parameters for cement-based materials is crucial for assessing the structural integrity of buildings. Both destructive (e.g., compression test) and non-destructive methods (e.g., ultrasound, computed tomography) are used to estimate Young’s modulus. Since ultrasound estimates the dynamic Young’s modulus, a formula is required to adapt it to the static modulus. For this formulas from the literature are compared. The investigated specimens are cylindrical mortar specimens with four different sand-to-cement mass fractions of 20%, 35%, 50%, and 65%. The ultrasound signals are analyzed in two distinct ways: manual onset picking and full-waveform inversion. Full-waveform inversion involves comparing the measured signal with a simulated one and iteratively adjusting the ultrasound velocities in a numerical model until the measured signal closely matches the simulated one. Using computed tomography measurements, Young’s moduli are semi-analytically determined based on sand distribution in cement images. The reconstructed volume is segmented into sand, cement, and pores. Young’s moduli, as determined by compression tests, were better represented by full-waveform inversions (best RMSE = 0.34 GPa) than by manual onset picking (best RMSE = 0.87 GPa). Moreover, material parameters from full-waveform inversion showed less deviation than those manually picked. The maximal standard deviation of a Young’s modulus determined with FWI was 0.36, while that determined with manual picking was 1.11. Young’s moduli from computed tomography scans match those from compression tests the closest, with an RMSE of 0.13 GPa.

## Introduction

The Young’s modulus *E* is together with the compressive strength $f_{{\text {c}}}$ the most important material parameter for structural design^1^. Also in the field of material research and discovery, determining the Young’s modulus is essential for evaluating the performance of new materials. Usually, the static Young’s modulus $E_{{\text {s}}}$ is used, which is commonly determined by compression tests of either cylindrical or cubic specimens. While the specimens have to be destroyed during the compression tests, the dynamic Young’s modulus $E_{{\text {d}}}$ can be determined in a non-destructive manner. Commonly used methods for this are ultrasound time-of-flight measurements or an impulse excitation together with the evaluation of the frequency response. Studies that use ultrasound or evaluate the frequency response for estimating $E_{{\text {d}}}$ can be for example found in^1–5^. $E_{{\text {s}}}$ and $E_{{\text {d}}}$ differ significantly from each other and $E_{{\text {d}}}$ is generally larger than $E_{{\text {s}}}$. According to Neville^6^, the reason for this discrepancy is that $E_{{\text {d}}}$ is measured at negligible stress levels in comparison to $E_{{\text {s}}}$ and, therefore, inelastic processes such as microcracks and creep are not introduced into the material. In contrast, mostly elastic effects dominate the dynamic elastic modulus. Neville^6^ states that due to this, the dynamic modulus is approximately equal to the initial tangent modulus $E_{{\text {0}}}$ of the static test. However, this view is challenged by Bastgen and Hermann^7^, where $E_{{\text {d}}}$ was experimentally determined as $1.15 \cdot E_{{\text {0}}}$. The authors do not give any reason for this difference. Another reason for the difference between $E_{{\text {s}}}$ and $E_{{\text {d}}}$, specifically for concrete, is the heterogeneity of the material^8^. Also the loading frequency of the test, which is quite different for ultrasound and the compression test, has an influence. For rocks, investigations of the frequency dependency of the Young’s modulus exist^9,10^. Here $E_{{\text {d}}}$ increases with the testing frequency. This can be described by viscoelasticity, which states that higher frequencies usually increase the equivalent stiffness. Furthermore, the acoustoelastic theory describes that the stress level has an influence on the measured ultrasound velocities and, therefore, the elastic constants^11^. Here a higher stress level, which is present in the static measurement, would lead to higher ultrasound velocities. Due to the complexity of the relation between $E_{{\text {s}}}$ and $E_{{\text {d}}}$ , there exists no analytical relation between the two. The existing relations in literature were determined empirically for a specific material.

In general, ultrasound $E_{{\text {d}}}$ often shows higher deviations when performing repeated measurements in comparison to the frequency response analysis (also called resonance testing)^2^. A reason for this is the heterogeneity in the material, which introduces a different response due to locally different material properties as shown in^6^. Empirical adjustment formulas exist in order to match $E_{{\text {s}}}$ and $E_{{\text {d}}}$ for concrete^13^. Lydon and Balendran^14^ proposed in 1986 the following relations between $E_{{\text {d}}}$ and $E_{{\text {s}}}$ for concrete

1 $$\begin{aligned} E_\text {s} = 0.83 \cdot E_\text {d}. \end{aligned}$$

In Marques et al.^3^ $E_{{\text {d}}}$ and $E_{{\text {s}}}$ were compared for different cement-based mortar mixes. They estimated the proportionality factor in Eq. (1) to be 0.837 using mortar with different fractions of sand, different ages, and ultrasound for determining $E_{{\text {d}}}$. This is very similar to the proportionality factor of Lydon and Balendran^14^, so the formula seems to be applicable for mortar as well.

Other researchers also incorporated further physical parameters like the bulk density $\rho$ into the formula to make it more generalizable. One of the most used empirical equations was introduced by Popovics^12^ and converted by Makoond et al.^4^ into GPa

2 $$\begin{aligned} E_\text {s} = \frac{446.09 \cdot E_\text {d}^{1.4}}{\rho }, \end{aligned}$$

where $\rho$ is given in $\frac{{\text {kg}}}{{{\text {m}}^3}}$.

Another method for determining the effective elastic material parameters of heterogeneous structures is by using imaging techniques like X-ray computed tomography (CT) scans. In the study conducted by Saenger et al.^15^, high-resolution CT scans were used to characterize sandstone. The scans had a voxel size of 1.6 $\upmu$m, and the resulting images underwent a segmentation procedure to distinguish between grains, pores, and the contact area between the grains. The specimens were put in an X-ray transparent pressure cell inside a CT scanner. Two distinct scans were carried out under different confining pressures: 1 MPa and 20 MPa. Notably, the application of higher pressure led to a reduction in porosity. With the segmented images, parameterized with values of the velocities of its constituent elements (quartz and air) obtained from the literature, the authors estimated the effective p- and s-wave velocities of the rock. This was achieved using the finite-difference method to solve the elastic wave equation. For comparison, the ultrasound velocities were also determined experimentally. Notably, the research revealed a significant discrepancy in the velocities estimated based on CT images and the experimentally determined ones. For the p-wave velocity in the 20 MPa experiment, this was more than 700 $\frac{{\text {m}}}{{\text {s}}}$. The authors attributed this mismatch to the absence of information regarding microcracks resulting from the applied loading, as well as the inability to resolve smaller pores in the images. To address these issues, the model was refined by adapting parameters in the contact zone of the grains, along with incorporating correction factors for the material properties of its constituents.

Andrä et al.^16^ present a study investigating various material properties of rocks using segmented CT images. Here, the hydraulic permeability, the electrical resistivity, and Young’s moduli are computed using different numerical solvers. The specimens were segmented into pores and rock material, with Young’s moduli of the rock phases derived from established literature values. Employing the finite element method with the Lippmann-Schwinger equation, the study determines the effective static elastic properties. The entire volume was subsampled into smaller volumes to estimate the precision of material parameter estimates. The estimated variation in the material parameters was attributed to the segmentation algorithm, the used numerical solver, and the subsamples investigated.

In this study, only ultrasound will be investigated for determining $E_{{\text {d}}}$. Further, it will be investigated if the formulas of Lydon and Balendran^14^ and Popovics^12^ are suitable for the conversion of $E_{{\text {d}}}$ to $E_{{\text {s}}}$. Conventional ultrasound measurement of concrete or mortar specimens involves performing two measurements in a through-transmission arrangement, employing p-wave and s-wave transducers. This conventional method relies on the manual identification of the onset of each wave mode to compute their respective velocities, which is also performed in this study. Moreover, this research integrates a sophisticated approach called full-waveform inversion (FWI). FWI enables the determination of both p-wave velocity $v_{{\text {p}}}$ and s-wave velocity $v_{{\text {s}}}$ through the utilization of a single measurement with a p-wave transducer. In Schmid et al.^17^ it was shown that the precision of the velocity determination with FWI is higher in comparison to the velocity determination with onset picking.

In this study, it is further explored if $E_{{\text {s}}}$ can be estimated using CT scans of mortar specimens. The method involves segmenting the CT scans and performing semi-analytical computations to derive the effective $E_{{\text {s}}}$. The specimens used in the study consist of mortar with varying sand proportions. The estimated Young’s moduli obtained through the CT and ultrasound-based analysis are then compared to those derived from conventional compression tests.

The paper is organized as follows: Section “Materials and methods” introduces the investigated mortar specimens. It subsequently presents the essential theoretical foundations and the measurement setup employed to estimate Young’s moduli through compression tests, ultrasound, and CT scans. The results are given in Section “Results and siscussion” and subjected to a discussion. Section “Conclusions” provides the concluding remarks.

## Materials and methods

### Investigated mortar specimens

As previously mentioned, four distinct mortar specimens, each with varying sand fractions of 20%, 35%, 50%, and 65% were examined. The mass fraction of the constituents was used to prepare the mixture. All specimens underwent testing after 28 days of manufacturing to ensure the hydration process was mostly finished and they reached almost their full strength^21^. A consistent water-cement ratio of 0.5 was maintained for all specimens to ensure similar microporosity in the cement and a consistent quality of the interfacial transition zone between the paste and aggregate, as recommended by Landis^18^. The mixture was initially dry-mixed for 30 seconds, after which water was added, and then wet-mixed for 60 seconds. The CEM I 52.5 N cement from HeidelbergCement was used. The sand consists mostly of quartz, with a grain size smaller than 2 mm.

The specimens were made according to DIN EN 12390-1^19^ and DIN EN 12390-2^20^. To fabricate the specimens, cylindrical molds with a diameter of 100 mm were used. The heights of the specimens were ranging from 190 to 197 mm. The variation in height was due to grinding the top and bottom surfaces of the cylinder to ensure reproducible ultrasonic coupling and even force transmission during the compression test.

**Figure 1:**
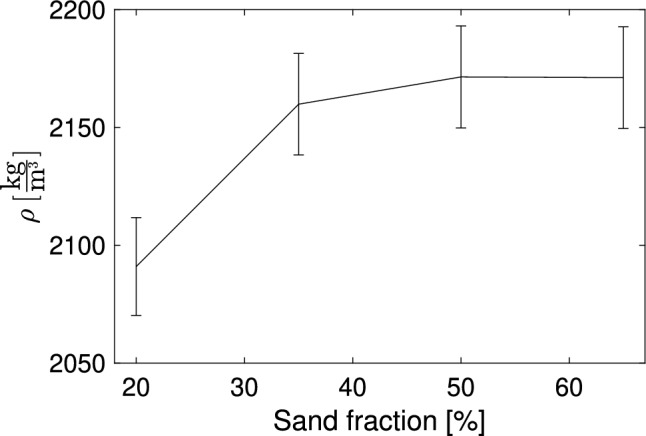
Estimated densities $\rho$ of the four specimens with different sand fractions. The standard deviation was computed through uncertainty propagation employing a Monte Carlo technique, utilizing the implementation of Klebba^22^.

The dimensions of each specimen were measured at four different positions, and the specimens were weighed four times with the scale zeroed between measurements. From the obtained values, the density $\rho$ of each specimen was calculated together with its standard deviation. The estimated densities are given in Fig. 1.

It can be seen that the relationship between sand fraction and $\rho$ is not linear.

### Determination of Young’s moduli through compression testing

The compression test was carried out in accordance with the DIN EN 12390-13^23^ standard to determine the value of $E_{{\text {s}}}$ of the material, which is a measure of the ratio of axial strain in a material under uniaxial load^6^. To estimate the Young’s modulus, it is essential to ensure that the stress applied remains lower than the compressive strength $f_{{\text {c}}}$ of the specimens, as specified by the DIN EN 12390-13 standard^23^. To address this requirement, an initial test was conducted on a second specimen manufactured concurrently with the same mortar mixture to estimate its $f_{\text {c}}$.

**Figure 2:**
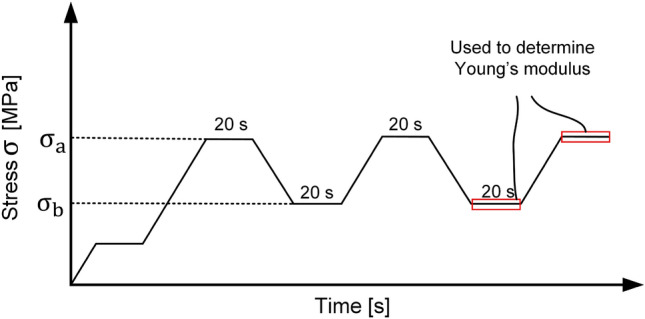
Testing cycle for the estimation of $E_{\text {s}}$ according to DIN EN 12390-13^23^.

Only specimens for which the Young’s modulus was estimated were considered for previous (ultrasound) or subsequent (CT) evaluations. The compression test procedure involves pre-stressing the specimen and then subjecting it to three cycles, during which a specific stress level is applied and subsequently released. This cyclic loading and unloading process is done to eliminate any creep effects, as detailed in the study by Makoond^4^. Each cycle was held for a duration of 20 seconds, and a graphical representation of this procedure can be seen in Fig. 2.

In each cycle an upper stress level $\sigma _{\text {a}}=\frac{f_{\text {c}}}{3}$ and a lower stress level of $\sigma _{\text {b}}=0.15 \cdot f_{\text {c}}$ were applied. For measuring the stain $\epsilon$ two vertical strain gauges were used and the result was averaged. The so-called chord or secant modulus is calculated in the third cycle as

3 $$\begin{aligned} E_\text {s} = \frac{\sigma _{\text {a}}-\sigma _{\text {b}}}{\epsilon _{\text {a}}-\epsilon _{\text {b}}}. \end{aligned}$$

Hereby, the stress and strain in regions marked in Fig. 2 as red are averaged. All tests were done on a Walter+Bai 600 kN compression test machine.

### Ultrasound for Young’s moduli estimation

There is no established standard for determining Young’s modulus using ultrasound for concrete or mortar. The measurements in this study are performed within a through-transmission setup, employing p-wave and s-wave transducers and corresponding receivers. In both measurements, the onsets of the receiving wave modes are manually picked.

Based on the so determined s- and p-wave velocities $v_\text {p}$ and $v_\text {s}$ and the density $\rho$ the dynamic Young’s modulus $E_\text {d}$ can be calculated as^24^

4 $$\begin{aligned} E_\text {d}= \frac{\rho \cdot v_\text {s} ^2 \cdot (3 \cdot v_\text {p} ^2 - 4 \cdot v_\text {s} ^2 )}{v_\text {p} ^2 - v_\text {s} ^2}. \end{aligned}$$

For the derivation of this formula please refer to Krautkrämer et al.^24^.

#### Measurement setup

The cylindrical specimens were measured in a through-transmission setup. The TiePie HS5 waveform generator and the Trek 2100 HF amplifier were utilized to excite the ultrasound wave. Further, the V101 p-wave transducer and receiver from Panametrics (Baker Hughes) was used for manual onset picking of the p-wave onsets and the determination of $v_{\text {p}}$ and $v_{\text {s}}$ using FWI. The diameter of the transducer is 25 mm. The transducer has a resonance frequency of 500 kHz. A square pulse with tow bursts and a frequency of 200 kHz was used as a excitation. The pulse was not perfectly square-shaped, and therefore not broadband. The lower excitation frequency was selcted to reduce attenuation effects within the material. This frequency was chosen after incrementally changing the excitation frequency and receiving the highest amplitude at 200 kHz. A transducer with a lower resonance frequency would be more suited here and lead to higher received signal amplitudes. The introduced wave mode is mostly a p-wave, which is also described in Schmid et al.^17^ for a similar setup.

Further measurements were conducted using an s-wave transducer and receiver, specifically the V150 from Panametrics (Baker Hughes) for the manual onset picking approach. This transducer has a resonance frequency of 250 kHz and was also excited at 200 kHz. We used the same excitation frequency as for the p-wave transducer in order to avoid influences due to different frequencies in the signals. The frequency spectra of a measurement with a p-wave and an s-wave transducer looked very similar, with both having the highest peak at 200 kHz. This similarity is due to two factors: using the same excitation frequency and the attenuation that removes the higher frequencies. The TiePie was controlled with a MATLAB script, and 100 measurements were conducted and averaged for each specimen-transducer configuration. All transducers were coupled using a shear wave couplant onto the specimens.

The system’s latency was estimated using a calibration specimen from Proceq. This calibration specimen has a known time-of-flight for the p-wave; thus, the velocity was known. The latency was estimated by conducting measurements on this specimen with the p-wave transducer in a through-transmission arrangement. The latency for the manual onset picking approach was estimated by picking the onset in this measurement. In the context of FWI, wave propagation in the specimen using the velocity of the calibration specimen was simulated. The latency was adjusted to match the simulated and measured waveforms. With manual onset picking, the latency was estimated to be 4.87 $\upmu$s, whereas with FWI it was 6.30 $\upmu$s.

#### Ultrasound modeling approach and full waveform inversion

Previously, Schmid et al.^17^ and Boxberg et al.^25^ introduced a method for estimating Young’s modulus for homogeneous materials with FWI. FWI, first introduced in geophysics, employs an iterative process to update a material model in a wavefield simulation by minimizing a cost function or misfit. The cost function quantifies the difference between the simulated and measured signal. With FWI material parameters such as wave velocities ($v_{\text {p}}$ and $v_{\text {s}}$), density ($\rho$), and, in certain cases, attenuation (Q-values) are updated. Typically, FWI is used to estimate heterogeneous material parameter fields using the adjoint method and gradient-based optimization^26^. However, it is important to note that our study exclusively focuses on homogeneous materials. In this study, the inversion is solely performed for the velocities, as density measurements can be easily obtained by measuring the dimensions and weight of a specimen. As a cost function, the least square-misfit $\chi _{\text {L2}}$ between the simulated $d_{\text {sim}}$ and measured $d_{\text {mes}}$ signal is used which is defined as^17^

5 $$\begin{aligned} \chi _{\text {L2}}= \frac{1}{2} \cdot \sum _{i=0}^{n-1} (d_{\text {sim},i}-d_{\text {mes},i})^2, \end{aligned}$$

with *n* being the number of samples in the signals. If the starting velocities are significantly different from the true velocities the $\chi _{\text {L2}}$ misfit exhibits local minima in the optimization procedure, due to the so-called “cycle-skipping”. Cycle-skipping occurs when individual hills and valleys in the signals of the two waveforms are phase-shifted with respect to each other^27^. Schmid et al.^17^ employed a different misfit function, introduced by Boehm et al.^27^ for ultrasound, known as the graph-optimal-transport misfit for this reason. This was necessary, due to the diverse range of materials they investigated in that study, which led to a significant difference between the starting model and the parameters the algorithm ultimately converges to. In contrast, this study deals with a much narrower range of velocities within the mortar specimens. Consequently, the $\chi _{\text {L2}}$ misfit is applicable. As a starting model, the measured velocities obtained through manual onset picking from the 50% mortar specimens were used for all optimizations. However, as a precaution to mitigate cycle-skipping, no gradient-based optimizer and instead the Powell algorithm^28^ was applied. The Powell algorithm conducts line searches in multiple directions during each iteration. The parameters yielding the lowest misfit in the search serve as the starting point for subsequent line searches in the next iteration. It’s worth noting that the Powell algorithm has relatively low requirements for the function to be optimized (e.g., it does not necessarily need to be differentiable). However, it may require a greater number of function evaluations compared to a gradient-based optimizer^29^. The SciPy’s implementation^30^ for the Powell algorithm was used.

**Figure 3:**
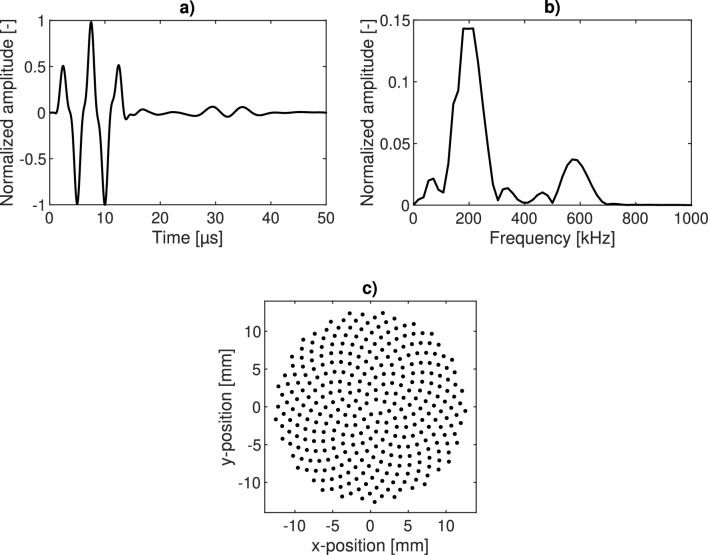
Modeling the approach of the ultrasound transducer. In (**a**), the excitation wavelet is depicted, along with its frequency amplitude spectrum in (**b**). In (**c**), the ensemble of point sources is visualized.

For FWI to work, the simulated and measured signals have to match as closely as possible. For that reason, it is important to model the excitation of the ultrasound transducer as accurately as possible^17^. In this study, the transducer was modeled in a “Fibonacci sphere”, which aims to distribute the point sources as evenly as possible over a circular area. This can be seen in Fig. 3c. All sources were firing at the same time. The wavelet was extracted from a measurement where the transducer and receiver were pressed directly onto each other (see Fig. 3a). The highest amplitude of the excitation is at 200 kHz as can be seen in the frequency spectrum of the excitation wavelet in Fig. 3b.

The wavefield simulations were conducted with the software Salvus, developed by Mondaic AG. Salvus leverages the spectral element method and is tailor-made for performing time-domain waveform simulations on the GPU, delivering highly efficient computational performance. The spectral element method represents a variant of the finite element method, distinguished by its diagonal mass matrix and employment of high-order piecewise polynomial basis functions (specifically, order 4 in this context). This approach offers high precision even when employing fewer degrees of freedom compared to traditional finite element methods. Consequently, it excels in simulating high-frequency wave propagation, particularly when a fine mesh is necessary^31^. All simulations in this study were carried out in 3D.

In the simulations of this study, attenuation was not taken into account. Attenuation arises from the scattering and absorption of ultrasonic waves, where scattering typically occurs at material interfaces (e.g., sand/cement), and absorption represents the conversion of the ultrasonic wave’s kinetic energy into heat^18^.

Given the simplified nature of the transducer model in comparison to Schmid et al.^17^, as well as the greater complexity of mortar material compared to metals, as used in Schmid et al.^17^, this study focuses on a shorter section of the signal. This approach was chosen because modeling inaccuracies tend to become more prominent at later times in the ultrasound signals. The attenuation effects were small in the regarded section of the signal.

For all wavefield simulations, a workstation featuring the subsequent hardware setup was employed: an NVIDIA GeForce RTX 3090 Ti GPU with 24 GB of GPU-RAM, an AMD Ryzen Threadripper PRO 3955WX, equipped with 16 cores, and 64 GB of RAM.

### Analytical estimation of Young’s moduli based on computed tomography images

All four specimens composed of different concrete mixtures were CT scanned. This process involved extracting a small portion, with a height and width ranging from 5 to 15 cm, from each cylindrical specimen after estimating the Young’s modulus in a compression test. The CT scans were carried out using a Yxlon Precision scanner, refurbished by Diondo. The voxel sizes from the scans ranged from 14.07 to 16.21 $\upmu$m.

The scanning parameters included an acceleration voltage of 100 kV, a current of 160 $\upmu$A, an integration time of 3220 ms, and a 0.5 mm aluminum prefilter. The detector employed had dimensions of 2048 x 2048 pixels. Subsequently, the projections were reconstructed using the Feldkamp, Davis, and Kress (FDK) algorithm^32^.

#### Segmentation and porosity calibration

**Figure 4:**
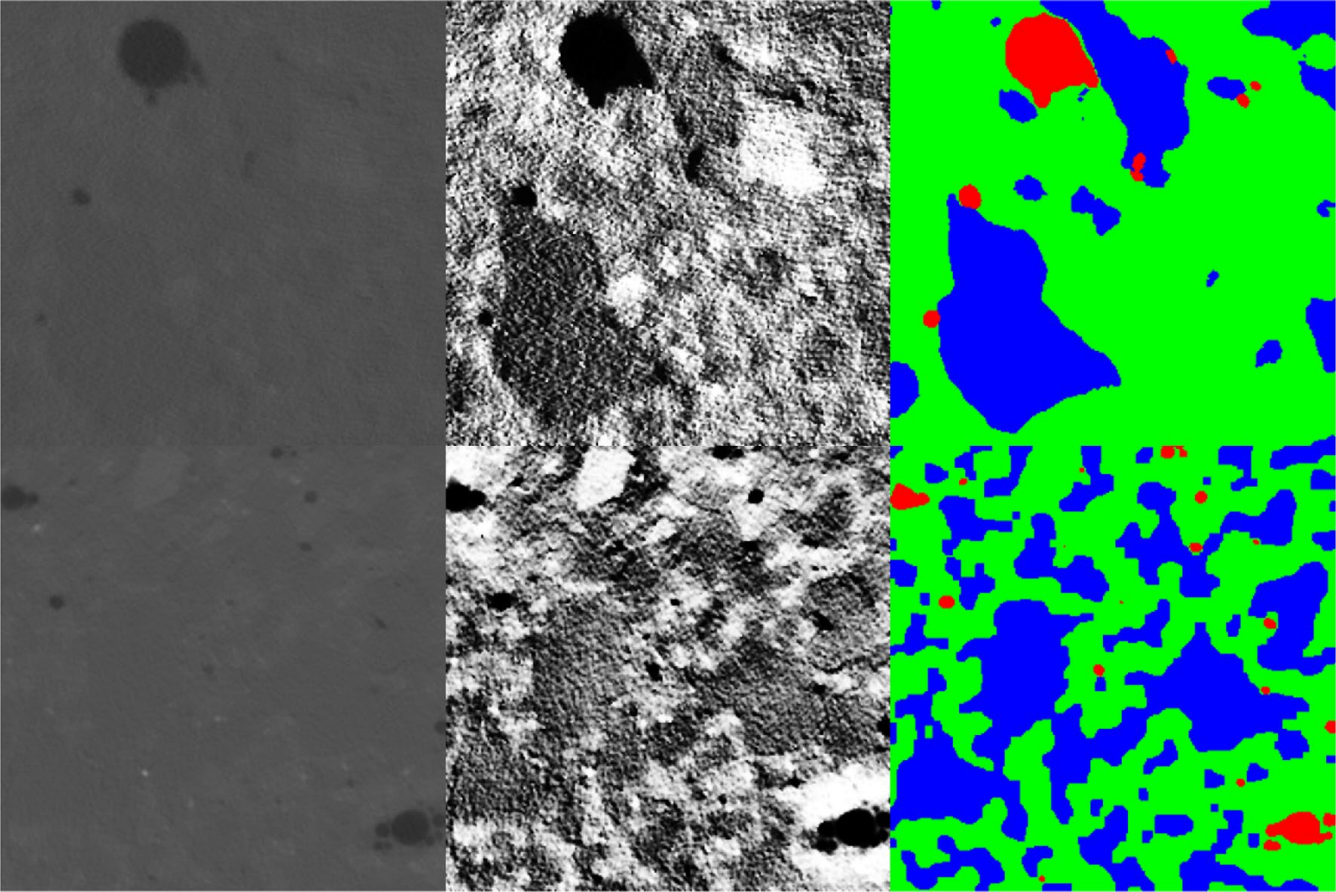
Unprocessed CT image on the left column, contrast-enhanced image in the middle column, and segmented image on the right column. The top row represents the 20% specimen, while the bottom row represents the 35% specimen.

In general, the contrast between the sand and cement region is low, due to the similar density and atomic number (e.g., both contain silicon) of the two constituents (see Fig. 4) left.

For that reason, in the next step, the image slices of the reconstructed volume underwent contrast enhancement by applying the contrast-limited adaptive histogram equalization (CLAHE) algorithm^33^ and global histogram equalization, implemented in MATLAB. A contrast-adapted image of the 20% and 35% specimens can be seen in the middle column of Fig. 4. The contrast-enhanced images were segmented into three classes: cement, sand, and pores (green, blue, and red, respectively).

The Segmentation was performed with Dragonfly from ORS and the inbuilt “segmentation wizard“. Hereby, single images are annotated manually, and a convolutional neural network (CNN), specifically a U-net, is trained on these images and used for inference on the whole image stack. The training was conducted with augmentation using rotation, flipping, and Gaussian noise. The cross-entropy loss was used as a loss function, and the Adadelta optimizer^34^ was applied to tune the learning rate during the training. Furthermore, early stopping was used in training. During the labeling process, attention was paid that the sand fraction within the labels matches the volume fraction of the sand within the specimen. Hereby, the labels sand or cement were manually adjusted such that the volume fractions match. The density of the constituents is needed to calculate the volume fraction based on the mass fraction. The density of sand was estimated to be 3100 $\frac{{\text {kg}}}{{{\text {m}}^3}}$ in previous investigations. Using this value, the porosity measured through the CT scans, and the estimated densities and volume of the four specimens, the density of cement was estimated as 1810 $\frac{{\text {kg}}}{{{\text {m}}^3}}$. With these densities, the volume fraction of sand was calculated as 26%, 38%, 47%, and 53%. The CT scans were annotated such that this fraction is also approximately reached in the annotation. In the end, the four specimens had a volume sand fraction of 27.28%, 36.12%, 46.22%, and 53.02% in their predictions. It should be mentioned that due to the low contrast, it is likely that there are inaccuracies in the annotations and predictions.

**Figure 5:**
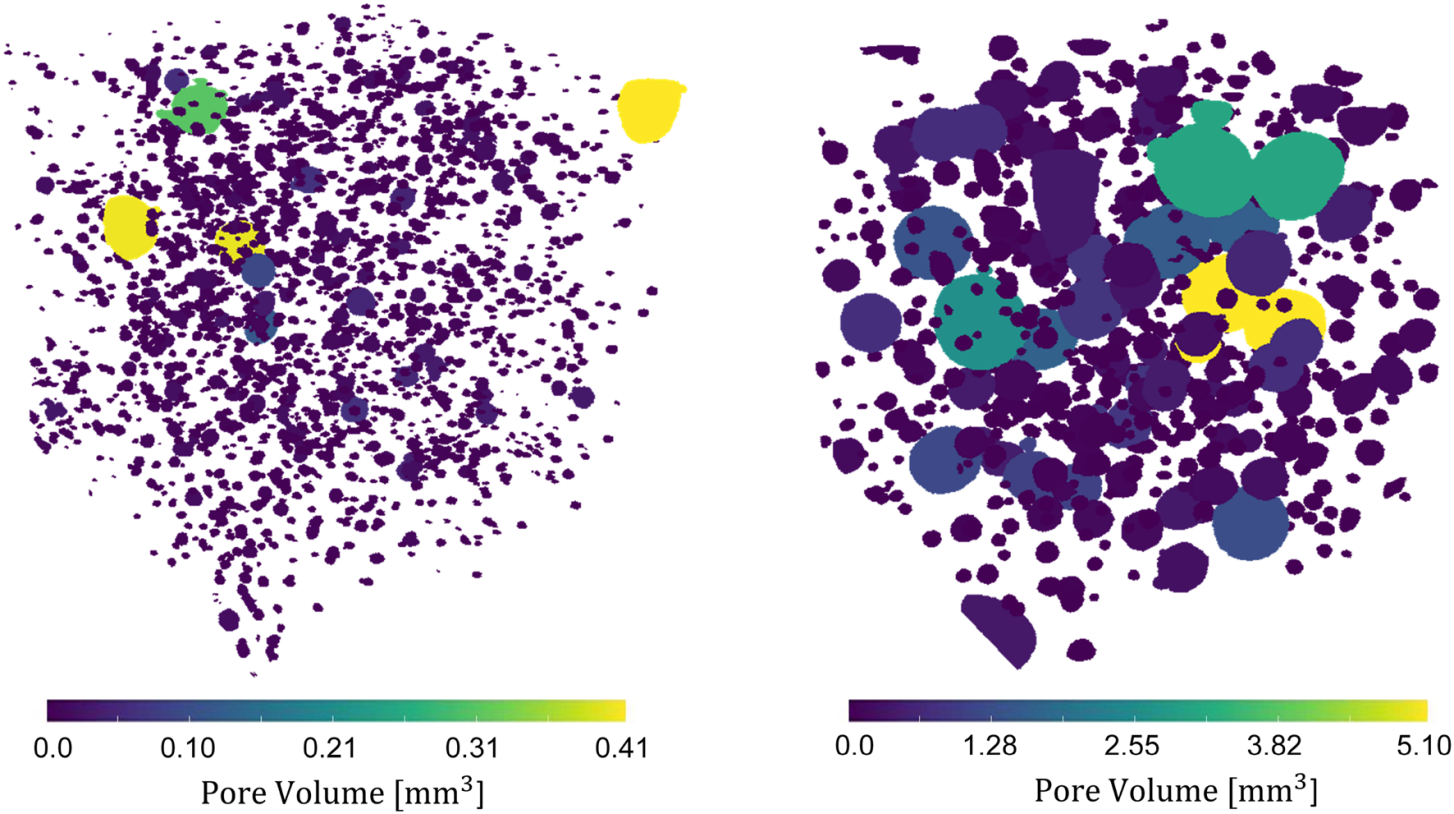
Segmented porosity in the CT scans of the 20% specimen (left) and the 65% specimen (right).

The porosity of the segmented CT scans was very different for the four specimens, increasing with a higher sand proportion. The estimated porosity for the four specimens (20%, 35%, 50%, and 65%) was 1.37%, 1.78%, 3.57%, and 7.26%, which represents the air pores. This aligns with the non-linear relationship of the density over the sand fraction in Fig. 1. The segmented porosity of the 20% and 65% specimens is shown for comparison in Fig. 5. The estimated pores in the CT scans represent macro porosity.

Every segmented pore with a diameter below double the voxel size was not regarded as a pore and was added to the cement class since the CT scan’s resolution is likely insufficient for such small pores.

Mercury intrusion porosimetry (MIP) was used to estimate the porosity below that threshold, which represents the capillary pores. Mercury is forced to intrude into voids inside a material with this device. When pressure is exerted, the mercury initially occupies the larger voids. With the rising pressure, it gradually infiltrates into progressively smaller pores. From each mortar specimen, a small glass vial was filled with small granular samples obtained from a portion of the entire specimen, and this sample was subjected to analysis. In the MIP analysis, particles larger than double the voxel size of the CT scans were excluded since this porosity is captured in the CT scans. The estimated porosity ranged between 11.08% and 17.03% for the mortar specimens, with no clear trend over the sand fraction. Therefore, it is assumed that the variation is due to the small sampling region within the specimen or measurement noise. The mean of these measurements with its standard deviation $13.18\% \pm 3.34\%$, will be used to calibrate the micro- and nanoporosity of the cement in the simulation. The macro porosity is already contained within the segmented images.

#### Multiscale modeling

The Young’s Modulus can also be computed by material modeling. To this end, a representative elementary volume (REV) of mortar is defined by specifying the spatial distribution and the geometrical and material properties of the constituents i.e. sand particles, pores, and hardened cement paste. The effective properties of this REV can be computed through homogenization using either analytical or computational approaches^35^. If multiple geometries over a wide range of scales are involved, REVs are specified at each scale and homogenization is performed sequentially.

**Figure 6:**
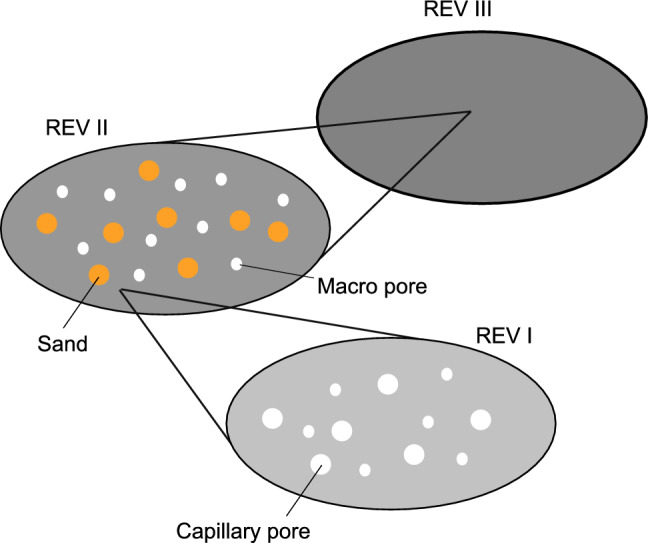
Illustration of the multiscale homogenization model.

The Young’s modulus of mortar can be computed by constructing a multiscale analytical model as follows (see Fig. 6): At the smallest scale of observation (REV I), the capillary pores, whose volume fraction is measured using MIP, are modeled as spherical zero-stiffness inclusions. These inclusions are assumed to be embedded in a cementitious material (without capillary pores). The capillary pores and the surrounding cementitious material together can be homogenized using the Mori-Tanaka scheme^36^ to obtain the Young’s modulus of the homogeneous material at a larger scale of observation (REV II), i.e., hardened cement paste. At this observation scale (REV II), the sand particles and air pores, also assumed to be spherical in shape, are embedded in the homogeneous hardened cement paste matrix (whose properties were just computed using homogenization). This REV, characterized by sand and air inclusions that are embedded in the hardened cement paste matrix, is also homogenized using the Mori-Tanaka scheme to obtain the Young’s modulus of mortar at the next larger scale of observation (III).

It must be noted that the described multiscale analytical model can be used to compute the Young’s modulus of mortar by specifying the properties of the constituents and their volume fractions. However, in our case, only a limited amount of information is available. While data corresponding to the volume fractions of the constituents is available from CT (sand, hardened cement paste, pores) and MIP (capillary pores), the material properties (Young’s modulus) of sand and the cementitious material are not known. To compute these quantities, the multiscale model in conjunction with least-squares minimization is used to downscale the mortar properties (obtained from compression tests) to the scale of the constituents. This is equivalent to inverting the multiscale model. Three measurements of the mortar Young’s modulus are used for identifying the following two unknown Young’s modulus: $E_{\text {sand}} = 121.59 \,\text {GPa}$ and $E_{\text {cm}} = 19.906 \,\text {GPa}$. The value $E_{\text {cm}}$ corresponds to the Young’s modulus of the cementitious matrix without capillary pores in scale (REV I). It must be noted that two material properties at two different scales have been computed using this inversion procedure. For a capillary porosity of $13.18 \% \pm 3.34 \%$ obtained from the MIP measurements, the Youngs modulus of hardened cement paste is obtained from homogenization as $15.26 \pm 1 \text {GPa}$. These values are used as input in the multiscale model to perform a forward/upscaling computation to estimate the stiffness of mortar.

## Results and discussion

### Evaluation of the ultrasound results

**Figure 7:**
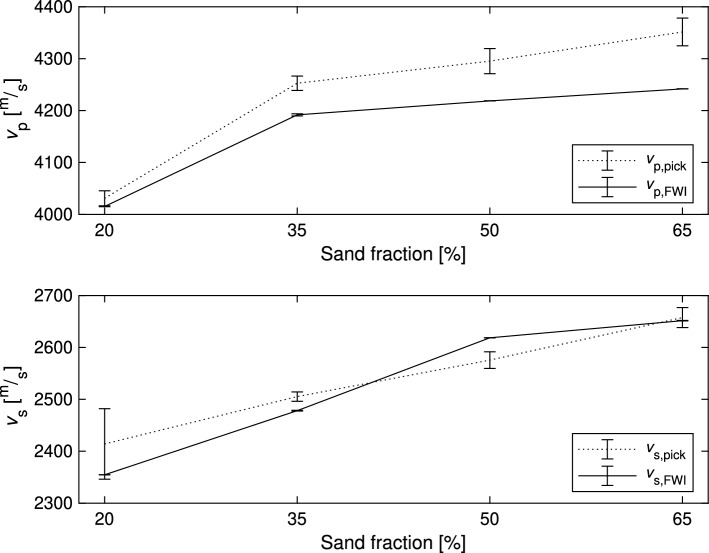
Estimated ultrasound velocities $v_\text {p}$ and $v_\text {s}$ for the four specimens with different varying sand fractions. The standard deviation was estimated using a Monte Carlo approach and the implementation of Klebba^22^. For the FWI approach the standard deviations are relatively small in comparison to manual onset picking.

Figure 7 presents the estimated ultrasound velocities obtained through two distinct methods: manual onset picking and FWI. In the case of manual onset picking, four different inspectors independently determined the velocities based on the signals acquired from the s- and p-wave transducers. The variations in their estimations are reflected in the standard deviation depicted in the plots. Here, the deviations in the height measurements of the specimens are also considered.

FWI was also performed four times, with the initial values for both velocities being adjusted within a range of $\pm 50 \, \frac{{\text {m}}}{{\text {s}}}$ from their starting value. Also, the deviation in the height of the specimen was regarded in the calculation of the overall standard deviation.

As the sand fraction increases, both velocities increase. However, it is noteworthy that there are disparities between the velocities estimated using FWI and those obtained through manual onset picking. Moreover, the standard deviations associated with the velocities estimated via manual onset picking are notably higher.

A plausible explanation for this discrepancy lies in the signal path within FWI. The signal undergoes a longer propagation path through the material due to reflections and mode conversions occurring at the boundaries of the cylindrical specimen. Consequently, local heterogeneities within the material have a reduced impact on the estimated values. It is important to recognize that local variations in material properties primarily contribute to the inherent uncertainties in ultrasound time-of-flight measurements within cement-based materials, as stated by Philleo^8^.

**Figure 8:**
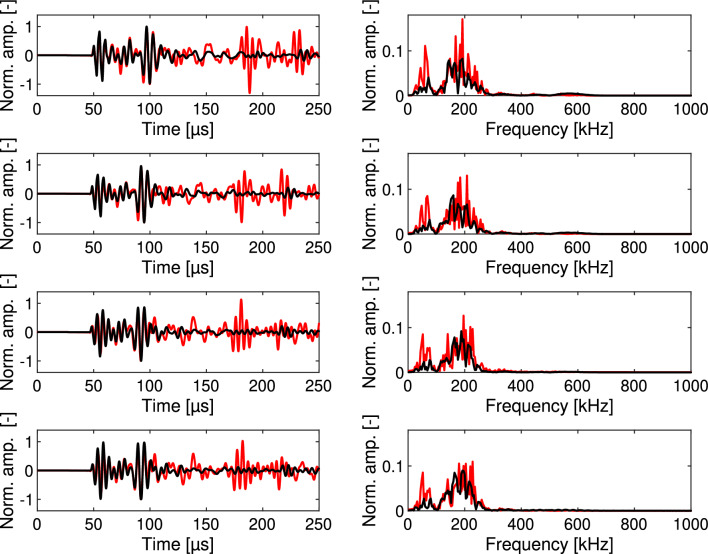
Simulated (red) and measured (black) ultrasound signals using the p-wave transducer, displayed from top to bottom in increasing sand proportions, ranging from 20% to 65%. The corresponding frequency amplitude spectra are visualized on the right.

Wavefield simulations were executed using the velocities estimated through FWI to assess the correspondence between the simulated waveforms and the measured ones. In Fig. 8, the simulated signals are plotted together with the measured signals using the p-wave transducer. Starting from the top and descending, the signals from specimens with sand fractions of 20%, 35%, 50%, and 65% are presented on the left.

Observably, the initial two wave packets align closely for the first arrival. However, a significant disparity in amplitude exists between the measured and simulated signals later in the signals, primarily due to neglecting attenuation. For that reason, the signals were considered only up to 150 microseconds for the FWI procedure.

The frequency spectra of the signals on the right reveal a decline in higher-frequency content (specifically at 600 kHz) in the measured signals as the sand fraction increases. This phenomenon is attributed to increased scattering resulting from the larger number of material interfaces (specifically between sand and cement)^37^. Furthermore, as the sand fraction increases, the direct p-wave, constituting the first arriving wave package, exhibits an earlier arrival time. The second wave package, as described by Schmid et al.^17^, corresponds to a p-s-p-wave, which converts from a p-wave to an s-wave and then back to a p-wave. It holds the information about the s-wave velocity.

### Comparison of the estimated Young’s moduli

**Figure 9:**
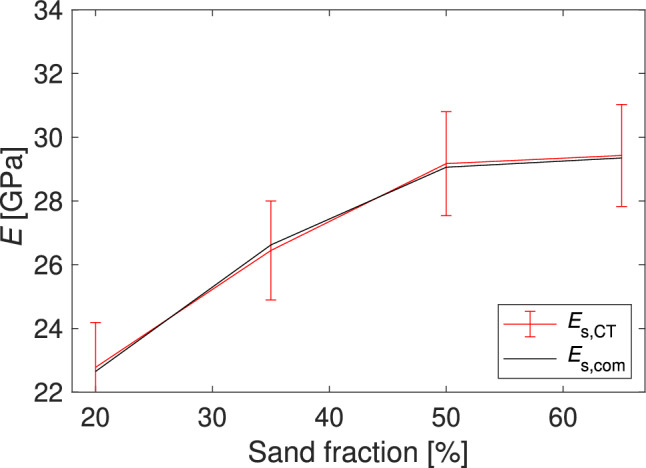
Estimated Young’s moduli $E_\text {s,CT}$ using the CT data.

For the estimation of the static Young’s moduli based on CT scans $E_\text {s,CT}$, the Young’s moduli of the constituents were at first determined using inverse homogenization. The estimated $E_\text {s,CT}$ are also given in Fig. 9 and Table 1. It can be seen that $E_\text {s,CT}$ and $E_\text {s,com}$ match well.

**Table 1:** Estimated Young’s moduli of the mortar specimens.

	$E \, 20\%$ [GPa]	$E \, 35\%$ [GPa]	$E \, 50\%$ [GPa]	$E \, 65\%$ [GPa]	RMSE [GPa]
$E_\text {s,com}$	22.65	26.62	29.06	29.35	–
$E_\text {s,CT}$	$22.78 \pm 1.41$	$26.45 \pm 1.55$	$29.17 \pm 1.63$	$29.43 \pm 1.60$	0.13
$E_\text {d,pick}$	$29.74 \pm 1.11$	$33.47 \pm 0.38$	$35.13 \pm 0.47$	$36.88 \pm 0.52$	6.91
$E_\text {d,FWI}$	$28.70 \pm 0.28$	$32.67 \pm 0.32$	$35.34 \pm 0.35$	$36.00 \pm 0.36$	6.26
$E_\text {d,pick}$ Lydon	$24.69 \pm 0.92$	$27.78 \pm 0.31$	$29.16 \pm 0.39$	$30.61 \pm 0.43$	1.33
$E_\text {d,FWI}$ Lydon	$23.82 \pm 0.23$	$27.11 \pm 0.27$	$29.33 \pm 0.29$	$29.88 \pm 0.30$	0.70
$E_\text {d,pick}$ Popovics	$24.65 \pm 1.30$	$28.15 \pm 0.52$	$29.96 \pm 0.63$	$32.08 \pm 0.70$	1.91
$E_\text {d,FWI}$ Popovics	$23.45 \pm 0.40$	$27.21 \pm 0.46$	$30.21 \pm 0.51$	$31.02 \pm 0.53$	1.13
$E_\text {d,pick}$ Lydon adapt.	$24.10 \pm 0.90$	$27.12 \pm 0.30$	$28.47 \pm 0.38$	$29.89 \pm 0.42$	0.87
$E_\text {d,FWI}$ Lydon adapt.	$23.26 \pm 0.23$	$26.47 \pm 0.26$	$28.64 \pm 0.28$	$29.18 \pm 0.29$	0.39
$E_\text {d,pick}$ Popovics adapt.	$23.74 \pm 1.25$	$27.11 \pm 0.50$	$28.85 \pm 0.61$	$30.90 \pm 0.68$	0.98
$E_\text {d,FWI}$ Popovics adapt.	$22.58 \pm 0.38$	$26.20 \pm 0.45$	$29.09 \pm 0.49$	$29.87 \pm 0.51$	0.34

The standard deviation was calculated using a Monte Carlo approach and the implementation of Klebba^22^ for the dynamic Young’s moduli. For the $E_\text {s,CT}$ repeated simulation results with varied porosity, and cement and sand Young’s moduli, were used. The RMSE between the Young’s moduli was estimated in reference to the compression test results.

The standard deviation in the estimated cement Young’s modulus was used with the deviation in the porosity for the forward calculation, resulting in the standard deviation of the mortar. The root-mean-square error (RMSE) between $E_\text {s,CT}$ and $E_\text {s,com}$, measured by the compression test, is the lowest of all investigated approaches. The RMSE between the two vectors *x* and *y* of the lenght *n*, which are in our case $E_\text {s,com}$ and $E_\text {d,~}$, is defined as

6 $$\begin{aligned} RMSE= \sqrt{\frac{1}{n}\cdot \sum _{i=0}^{n} (x_i-y_i)^2}. \end{aligned}$$

**Figure 10:**
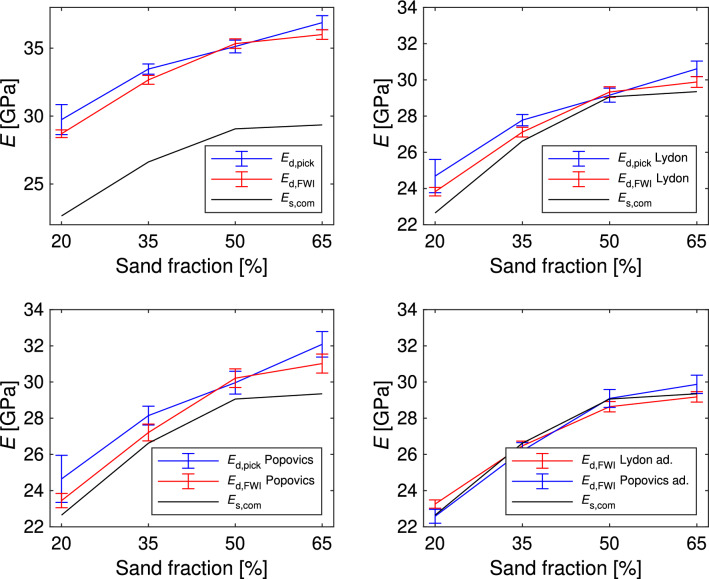
Comparison of estimated static ($E_\text {s}$) and dynamic ($E_\text {d}$) Young’s moduli for mortar specimens. The top-left plot shows the ultrasound and compression test results. The top-right and bottom-left subplots employ Eqs. (1) and (2) to rescale the Young’s moduli estimated with ultrasound. These equations were adapted to the data, and the outcomes are illustrated in the bottom-right plot.

Figure 10 illustrates the Young’s moduli estimated via ultrasound and compression tests. In the top-left plot, dynamic Young’s moduli ($E_{\text {d}}$) obtained through manual onset picking, and FWI are compared against the static Young’s moduli ($E_{\text {s}}$) determined by the compression test. As expected, a noticeable shift between $E_{\text {d}}$ and $E_{\text {s}}$ is evident. The trend in $E_{\text {s}}$ is more accurately captured by the dynamic Young’s modulus estimated with FWI, denoted as $E_{\text {d,FWI}}$. Further, the standard deviation is higher for the dynamic Young’s moduli estimated through manual onset picking than those estimated via FWI.

The top-right, bottom-left, and bottom-right subplots present different approaches to determine $E_{\text {s}}$ from $E_{\text {d}}$. In the top-right subplot, the Eq. (1) from Lydon and Balendran^14^ was applied. In the bottom-left subplot, Eq. (2) by Popovics^12^, is utilized. Employing the Eq. (1) the moduli exhibit a closer alignment than with Eq. (2). Furthermore, both equations were adapted to this dataset. The results of the adjusted formulas are shown in the bottom-right subplot. For Eq. (1), the new proportional factor was recalculated as 0.81, compared to the original value of 0.83. In Eq. (2), the proportional factor in the numerator was estimated as 429.58 GPa, previously 446.09 GPa. Since the original formulas are derived for concrete, the adjustment of these formulas should give the reader an impression of how the formulas need to be changed for mortar. The generalizability of this is still to be validated.

Table 1 presents the estimated Young’s moduli and their associated standard deviations, which were determined using a Monte Carlo approach. Generally, it is observed that both the standard deviation and RMSE are smaller when FWI is employed than when manual onset picking is used. The RMSE values were computed by comparing all estimated Young’s moduli against the values obtained from the compression test for each method. Notably, the Young’s moduli determined using $E_{\text {d,FWI}}$ and transformed to static Young’s moduli using Eq. (1) exhibit the smallest RMSE when the formulas are not adapted to the data. However, the adapted formula from Popovics, Eq. (2), yields the overall lowest RMSE for the dynamic Young’s moduli.

## Conclusions

This study compared different methods for estimating Young’s moduli for mortar specimens with different sand mass fractions of 20%, 35%, 50%, and 65%. At first, dynamic Young’s moduli were estimated using ultrasound. Hereby, the first arrivals of the p-waves and s-waves were determined by manual onset picking with two separate measurements using a p-wave and a s-wave transducer. With the measurement using the p-wave transducer, the p-wave and s-wave velocities were also determined using full-waveform inversion. The information about the s-wave velocities stems from mode conversion on the boundary of the cylindrical specimens. Since the dynamic Young’s moduli are generally higher than the static ones, an adaptation formula is needed to match these two parameters. For this, the formulas of Lydon and Balendran^14^ and Popovics^12^ were used. After the ultrasound measurements, static Young’s moduli were estimated with a compression test. Further, parts of the specimens were CT scanned and segmented. Static Young’s moduli were estimated using a semi-analytical approach based on the segmented reconstructed volume. For this, the sand and cement material parameters were first determined by an inversion procedure.

The main findings of this study can be summarized as follows:

- The velocities and Young’s moduli determined with full-waveform inversion shows a smaller standard deviation than the one determined with manual onset picking. With full-waveform inversion, only one measurement is needed.
- The dynamic Young’s moduli determined with full-waveform inversion matches better the trend of the static one than the one determined with manual onset picking.
- Both the formula of Lydon and Balendran^14^ and Popovics^12^ are valid for matching the static and dynamic Young’s moduli, but might need some adaptations for the specific cement-based material that is used.
- A new approach for estimating the Young’s moduli of the constituents based on a CT scan was introduced. Further, with the estimated material parameters, the effective Young’s moduli of the mortar specimen were calculated, which matches well with the measured one by the compression tests.

In future studies, we want to extend the experimental design to different mortar or concrete mixtures and also include the comparison of vibration-based methods with eigenfrequency evaluations for Young’s modulus estimation. Further, a joint inversion on the static and dynamic elastic properties of the constituents using a CT scan could enhance the accuracy of the estimated material parameters.

## Data Availability

The datasets generated and analyzed during the current study are available from the corresponding author on reasonable request.
